# Public and Private Nursing Institutions

**Published:** 1887-04-02

**Authors:** 


### Public and Private Nursing Institutions.

By a Hospital Physician.

By most medical men and educated nurses Miss Manson's paper will be considered a very unusual contribution to come from a lady's pen. The language is terse, and yet classically good. The principles are clearly defined; the subject is almost exhaustively treated. It is, moreover, studiously impartial, and, I should imagine, written in the hope rather of ventilating than of dogmatising on a most difficult question. I venture to congratulate Miss Manson on adding another to the many laurels she has already won, and on making yet another attempt to benefit both the public and the nursing profession. With regard to some of the articles in reply, anyone who respects that noble profession must keenly regret that they have been written or published; for the lamentable lack of courtesy in their tone is only equalled by the amusing absence of grammatical English in their composition.

With regard to the views of "A Barrister," I should like to say a few words. Far be it from me to hint that, as on his own showing, his experience of private nursing institutions is small, so his knowledge of the whole subject must be smaller. But he "is entirely opposed to our hospitals letting out nurses on hire." Why? Because it is a "commercial speculation." He will, I hope, pardon me for telling him that it is nothing of the kind. The system he is trying to bolster up is of course a "commercial speculation"; and it is just for that very reason that it is so manifestly unfair to patients and to nurses. But if "A Barrister" understood the question, he would see that in the case of hospitals taking up the work, first of all there would be no "profits" at all till all the expenses were paid; then, that the expenses include costs of management, which therefore would not be defrayed out of the "charity moneys." Further, that out of the profits larger wages could, and most certainly would, be paid to nurses than they would get from any "commercial speculation." Finally, if there were any surplus, it could be used to create and support a Nurses' Pension Fund.

But none of Miss Manson's critics have gone to the root of the question. Why did the Westminster Hospital, The Hospital for Women in Soho, the London Hospital, Guy's, and St. Bartholomew's, and others, begin to send out private nurses? Why are all the remaining hospitals in London and the provinces commencing, or declining to commence, the same system? Simply because the question is the eternal one of supply and demand. Medical men to-day demand a better nurse than they did ten, fifteen, or twenty years ago. The public demand more nursing and better nurses than they did at the same distance of time. One or two nursing institutions have done their work conscientiously to the patients, and with justice to their nurses. They have succeeded and prospered, and will, I trust, long continue to prosper. But the demand exceeds *their* powers to supply. And it must not be forgotten that nurses are of infinitely higher tone as well as training now than formerly. They know their value, and good nurses will not go to second-rate institutions, however your correspondents may blink the fact. Miss Manson did not say all this, but she has evidently realised at once that a new order of things has arisen; and she is evidently anxious to draw from the march of events benefit to her own profession. Finally, the tendency of the age is towards specialism; and just as the public insist daily more and more on their physicians being specialists, so will the medical profession more and more insist on their nurses being specially chosen for the case they have to nurse. And this, as Miss Manson so lucidly pointed out, can best be done in an institution where the nurses are numerous, and the exact capabilities of each accurately known and tested for years.

### "A Lady Superintendent of a Provincial Nursing Institution" on Miss Manson's Reply.

Miss Manson, in her remarks this week, says:—"The opinion of a Lady Superintendent of a Provincial Nursing Institution would *possibly* have been of more value had she seen *my* paper." I have at last read it, and do not see anything to alter my opinion of the difference between nurses going to private cases from a hospital or from a nursing institution. I quite agree with her as to the time of training, and have named this before, and must emphatically protest against nurses being called trained after a few months in hospital. I cannot see that it will benefit hospitals, in a pecuniary point of view, to send out private nurses except in the way I have seen it done, which is detrimental to the hospital patients, the private patients, and the nurses. The hospital patients perhaps suffer least, as the nurses have to do double duty. At private nursing institutions, when the nurses are all engaged, the Lady Superintendent says, "None in." In hospital nursing institutions no case is refused. The consequence is the wards are cleared of nurses. The Lady Superintendent says to the sister, "I want your nurse for private nursing." The sister, trained to obedience, has to submit; and I have heard a patient under these circumstances counting up ten nurses attending in a fortnight, as the nurses were constantly taken away. Could we organise a system of health, so that the rich would be ill at one time, the poor at another, it might work; or could it be, as "Barrister" says, that the *farm nurses* were *in fact* kept entirely separated from ward nurses, one could not say anything against it; but I feel assured that any sister who has been connected with a hospital that sent out private nurses will bear me out in the assertion, not only of the wards being left with insufficient hands, but nurses have been sent out with insufficient training, less than any nursing institution would think of.

I must emphatically deny Miss Manson's statement that the best nurses are always retained on the staff, ranging from Lady Superintendent to Staff Nurse. It is well known that rarely any lady trained in any hospital, or holding any post in it, is selected as lady superintendent; nor am I sure but this may be right, although hardly appearing fair. I have happened to see one of the few instances where a sister was appointed lady superintendent, and it did not work well, and was not surprised to hear a resolution was made that it should not be repeated. Miss Manson acknowledges she knows of six nurses with excellent qualifications who have left her and gone to nursing institutions. Might they not be the only six who would be accepted? How many may have gone from other hospitals? But evidently Miss Manson believes no nurse can be properly trained who has not been under her personal supervision. What a shock to the profession, patients and nurses, to find the announcement in The Hospital that she is about to enter the bonds of matrimony, so that her services must be lost! Shall we ever be able to get a trained nurse again? No doubt Miss Manson's ideas will enlarge when she emerges from the hospital walls. We all know in hospital work how our minds get narrowed into one groove inevitably.

Miss Manson asks Mrs. Bedingfeld for what philanthropic object these institutions exist. I can only answer for our own, which was started for the purpose of providing district nurses gratis for the poor; nurses at reduced rate when necessary; lending invalid appliances, and taking patients who required extra medical attendance and nursing, which are supplied at a very moderate rate. It was hoped the private nurses' fees would bring in a surplus to aid the district work, but there has been some disappointment in this, and the subscriptions have had to be continued. The time may come when the philanthropic objects have to be enlarged,—should Miss Manson, by her sweeping assertion, not be able to annihilate Private Nursing Institutions. I can quite believe that applications are more numerous for parish or district than Miss Manson or anyone else can supply, as the work is so heavy, disagreeable, and underpaid.

### Reply of "A Private Nurse."

Being the member of "The London Association of Nurses," to whom Miss Manson refers as having had three months' surgical training in St. Bartholomew's Hospital, I should like to state the circumstances under which my application was made.

Previous to joining the above Association, I had received two years' hospital training for medical work, and was accepted by the lady superintendent as a purely medical nurse. During the seven years I have worked in connection with the Association, I have been constantly attending medical cases—two were of six months' duration, and one of fifteen months'; and my one reason for applying for any further training was, that I thought some little insight into surgical nursing would be of service to me in my general work, where one never can tell what may be required, even from a medical nurse.

I should also like to say that the nurse who joined our ranks on February 1st, 1887, and was accepted without any special recommendation from Miss Manson, is the sister of one of our most valued members, and has had, to my personal knowledge, five years' hospital experience; she also held such high testimonials from doctors and others, that I presume no further references were deemed necessary.

